# An early warning model of type 2 diabetes risk based on POI visit history and food access management

**DOI:** 10.1371/journal.pone.0288231

**Published:** 2023-07-26

**Authors:** Huaze Xie, Da Li, Yuanyuan Wang, Yukiko Kawai

**Affiliations:** 1 School of Computer Science and Technology, Hainan University, Haikou City, Hainan Province, China; 2 Faculty of Engineering, Fukuoka University, Fukuoka City, Fukuoka State, Japan; 3 Graduate School of Sciences and Technology for Innovation, Yamaguchi University, Ube City, Yamaguchi State, Japan; 4 Division for Frontier Informatics, Kyoto Sangyo University, Kyoto City, Kyoto Prefecture, Japan; 5 Cybermedia Center, Osaka University, Ibaraki City, Osaka Prefecture, Japan; TU Wien: Technische Universitat Wien, AUSTRIA

## Abstract

Type 2 diabetes (T2D) is a long-term, highly prevalent disease that provides extensive data support in spatial-temporal user case data mining studies. In this paper, we present a novel T2D food access early risk warning model that aims to emphasize health management awareness among susceptible populations. This model incorporates the representation of T2D-related food categories with graph convolutional networks (GCN), enabling the diet risk visualization from the geotagged Twitter visit records on a map. A long short-term memory (LSTM) module is used to enhance the performance of the case temporal feature extraction and location approximate predictive approach. Through an analysis of the resulting data set, we highlight the food effect category has on T2D early risk visualization and user food access management on the map. Moreover, our proposed method can provide suggestions to T2D susceptible patients on diet management.

## 1 Introduction

Diabetes is a chronic disease characterized by high levels of blood sugar and body mass index (BMI). According to the centers for disease control and prevention (CDC) data, more than 37 million Americans have diabetes, and 90–95% of them have type 2 diabetes (T2D). T2D occurs when the body becomes resistant to insulin or doesn’t produce enough insulin. A high-fat diet is a direct cause of an increased risk of diabetes, as it can lead to obesity and insulin resistance [1]. According to the American Diabetes Association (ADA), the effectiveness of medical nutrition therapy (MNT) in the management of diabetes has been well established. Dietary structure management is currently the most effective method to control BMI and glycemic indices in T2D-susceptible patients [2]. The literature highlights the importance of regional food categories as explanations of dietary choices [3] and diet-related health outcomes, such as obesity and diabetes [4].

The goal of this study is to predict the risk of regional food categories, considering the dietary structure of individuals susceptible to T2D, as well as the food access in different urban facility areas. By combining the food choice factors and T2D risk visualization, we aim to increase awareness among consumers about early dietary management of T2D symptoms. The term “Food Access” refers to the decisions made by consumers regarding their food spending and diet. This encompasses the accessibility and affordability of food retailers, including factors such as food prices, travel time to shopping locations, and the availability of healthy food options. To this end, we try to map key categories of food access from T2D features to point-of-interest (POI) attributes on the map and predict the early susceptibility risk by POI visit frequency from social media check-in history.

In our previous research, we created knowledge graphs by extracting keyword relationships from T2D literature [5], and visualized the spatial features of POIs related to food access using map slices [6]. In this study, we aim to identify an appropriate methodology to count and analyze T2D food access categories, and to understand the significance of risk features on the map. Using geographic feature analysis of T2D diet structure, we classified 10 food access categories for structural feature separation of POI information on the map [7]. This analysis includes spatial heterogeneity: the difference in POI distribution reflected by T2D on time and space scales, discretized spatial data distribution: classification of POI features with the same category, and data-structured feature prediction: early regional predictions with T2D risk. To achieve these goals, we introduce spatio-temporal interaction features of POIs visited by social media users to discuss the T2D model of food access category decay in geospatial distance [8]. We concatenate the POI spatial vector and the temporal vector of Twitter users’ visits to POIs to create food access-related POI spatio-temporal visit features, including user visit frequency. Finally, we proposed a graph with long short-term memory (GLSTM) model to predict the relationship between the dietary structure of T2D and the distribution of POIs related to food acquisition categories on the map. This allowed us to obtain visualized risk results of early T2D-susceptible geographical areas.

Existing spatio-temporal POI structured feature prediction efforts typically focus on location recommendation or cases using auto-regressive integrated averaging, including dynamic disease propagation of the long short-term memory (LSTM) prediction model [9], and patient-level sequential modeling approach utilizing sequential dependencies to render a personalized prediction of the prescription efficacy [10]. However, an obvious limitation of these models is their inefficiency in training correlations between POIs and food access categories, where the mapping of multiple categories of data needs to be considered. To implement POI attributes’ training and category prediction, we construct a graph structure between user visit categories and POI attributes to search the T2D food access category for relevant POI attributes and use the mapping relationship to construct a graph convolution network (GCN) model. The main contributions of this study are as follows,

1. A new perspective: an early T2D risk prediction model using geographical information along with food access categories is proposed.
2. A graph structure-dependent relationship processing of map tiles is completed, which significantly contributes to regional T2D risk analysis.
3. We propose a graph neural network model for POI attributes’ training, which involves T2D category mapping and social media data evaluation.
4. We propose a temporal-based category mapping of the early T2D dietary structure risk prediction method.

## 2 Related work

### 2.1 T2D case category extraction

The structural level and complexity of the diabetes medical characteristics are described by data in different dimensions, such as the food access categories are primarily affected by BMI indicators and blood glucose levels. Therefore, the dietary structure category extraction map to the POI attributes with geographic information is a complicated process. Specifically, POI attributes extraction is depended on the spatial diversity called spatial heterogeneity which represents a general attribute of the inequitable distributions in spatial issues. A study summarizes the majority of the literature concerned with machine learning and data mining techniques applied for the prediction of diabetes and associated challenges [11]. Related studies conclude that machine learning-based methods are efficient, credible, and accurate in T2D-related category extraction tasks. Similar to our study, a work [12] proposes an end-to-end neural framework, FATE, which is based on a tensor-based graph neural network (GNN), LSTM, and a mixture attention mechanism, which allows for (a) predictive explanations based on learned weights across different feature categories, (b) reduced network complexity, and (c) improved performance in both prediction accuracy and training/inference time.

For the T2D food access category map to inequitable distributions, POI attributes process on the map, and the statistics of placemark contribute the automatic counts method, which combines map feature extraction and classifiers. A work introduces the currently T2D-related available feature extraction algorithms and models for blood glucose level prediction, where the artificial neural networks and hybrid models show better results [13]. The spatial stratified heterogeneity analysis investigates the heterogeneity among various strata of explanatory variables by comparing the spatial variance within strata and that between strata [14]. A study proposed a recurrent neural network (RNN) model which is used to detect meal information with the smart sequential tableware data and recognize meal information with multi-instance learning [15].

On the other hand, extracting feature information from diabetes cases for risk prediction of susceptible populations is also reflected in disease management research. A work introduces the overview of graph neural network models apply to disease feature extraction and drug discovery [16]. A research employs the interpretable filter-based convolutional neural network (IF-CNN) prediction model and pet dog-smell sensing (PD-SS) algorithm to enhance the general plan of disease prediction from patients database, which can automatically predict diabetes from PIMA Indian diabetes datasets [17]. A label-efficient, weakly semi-supervised deep learning algorithm for EHR phenotyping (WSS-DL) is proposed to leverage the crucial phenotyping information contained in EHR features from unlabeled samples [18].

### 2.2 Time-series geographic information analysis

Considering the temporal POI attributes with social media visit frequency are utilized to evaluate the T2D food access category, we also focus on temporal iteration tasks. For T2D prediction, a patient-level sequential personalized prediction approach is proposed to quantify the relationship of the multiple T2D sequence records with prescriptions and efficacies [10]. A study introduces the temporal abstraction to predict mortality with heterogeneous EHR data, which is employed to transform the heterogeneous multivariate temporal data into a uniform representation of symbolic time intervals and discovered the frequent time intervals related patterns (TIRPs) [19]. For temporal clinical case, a study employs deep learning networks of convolutional neural network (CNN) with LSTM combination to automatically detect the abnormality, which diabetes is diagnosed by the analysis of heart rate variability (HRV) signals obtained from ECG signals [20].

Based on the LSTM module of data training models are applied to the temporal detection tasks. A study proposes an epidemic prediction model, and the results show that LSTM can improve the prediction results of the daily spread of the COVID-19 model [21]. A work explores the association between diabetes and Alzheimer’s disease, they contribute the dataset with the most relevant features and complete the classification using the proposed convolution graph long short-term memory (CGLSTM) [22]. And a work verifies the effectiveness of Bi-LSTM [23]. Compared with the traditional LSTM method, Bi-LSTM improves prediction accuracy. In addition, LSTM also has a good performance on time-based case analysis, they proposed the GLSTM-DTA for DTA prediction, which combined graph neural network (GNN) with LSTM to extract drug features and protein features, which is facilitating to capture of long-term dependencies in sequence [24]. In addition, the Graph LSTM addresses the limitations of sequential models, and also help to utilize the semantic correlation between cases and categories on the temporal [25].

## 3 Overview

**Fig 1:**
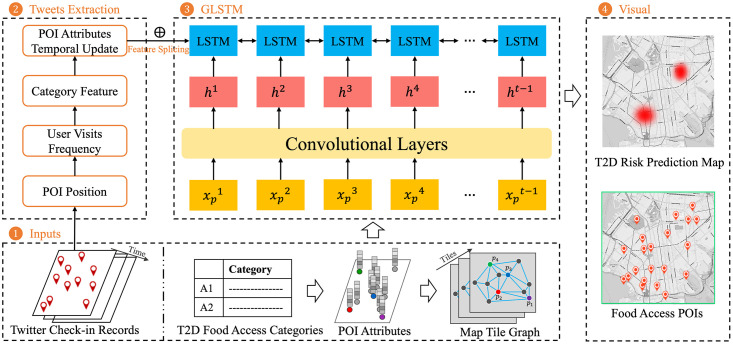
The overview of the GLSTM framework. Which contains the Data Input Block, Tweet Extraction Block, Graph Convolution with LSTM Block, and Output Visualization Block (on open map background: OpenStreetMap).

In this study, we aim to propose a map tile graph training framework to predict regional T2D risk and build a foundation for food access management. Food access management includes feature mapping from the T2D cases category to POI visit attributes at map tiles, and POI features temporal updating by users’ visit history records from social media. In Fig 1, we introduce the details of the proposed GLSTM framework, which is composed of four parts: an input block, a tweets extraction block, a GLSTM training model, and a visualization block. Considering personal information protection, the accuracy of visualized case geolocation is weakened as an ambiguous position.

### 3.1 Input block

We collect the food access category attributes of diabetes-diagnosed patients to extract the features of food access. A graph structure is constructed by nodes (food access relative POIs) and edge information (T2D food access categories). The training target in the next step is the relationship between the POI category and risk prediction on the map tiles.

### 3.2 Tweets extraction block

Twitter check-in data are collected from tweets with geolocation, and utilized to denote the user-visited frequency of related POIs. The category features are contributed by POI visit frequency and tweet attributes in the same map tile, where the dynamic user visit history on a time series is utilized to update the temporal POI attributes by the LSTM module.

### 3.3 GLSTM training model

GLSTM contains a graph convolution neural network and an LSTM module. The input map tile information is extracted as a feature matrix of each POI to denote regional T2D food access attributes. In addition, the LSTM module is utilized to model the time-series historical data for the weekly POI visit features of the food access categories. The GNN and LSTM are trained and optimized to compare the POI visit behaviors between category and Twitter users to predict the regional food access risk of T2D.

### 3.4 Visualization block

The visualization of high-risk areas for the T2D food access categories is defined by the region of user-visited POIs and the risk of case features. For food access management, our results will be used for food access selection and diet matching in T2D-susceptible populations.

## 4 Data collection

In this study, data collection is focused on the states of Florida, Georgia, Virginia, Pennsylvania, New Jersey, New York, and Massachusetts in the US. The main reasons why these states were selected are 1) The dense open data footprint (social media and open data) which is available (i.e. there are sufficient numbers of geotagged tweets, and OpenStreetMap is generally found to be more accurate in such densely populated urban areas [26]); 2) The majority of the data is available in English, which is necessary as we are only able to assemble a sufficient number of training samples in that language when developing the training model used to classify the different food access categories.

The T2D food access categories are defined based on a consensus regarding the dietary environment [7]: “low access stores”, “grocery stores”, “supercenters”, “club stores”, “convenience stores”, “specialized stores”, “full-service restaurants”, “fast food restaurants”, “school food service”, and “direct farm sales”.

In this study, we extract 100 map tile samples with POI attributes from OpenStreetMap. We utilize 73,068 pieces of POI attributes map to the T2D-related food access categories, and 198,377 tweets are utilized to evaluate the training model and predict the early risk on the map tile. We collect geo-data from OpenStreetMap to represent the spatial characteristics of each POI, and geotagged tweets are analyzed and utilized to represent the food access categories from user visit history. When collecting data for this study, we complied fully with the terms and conditions for the data sources.

### 4.1 Data preprocessing

**Fig 2:**
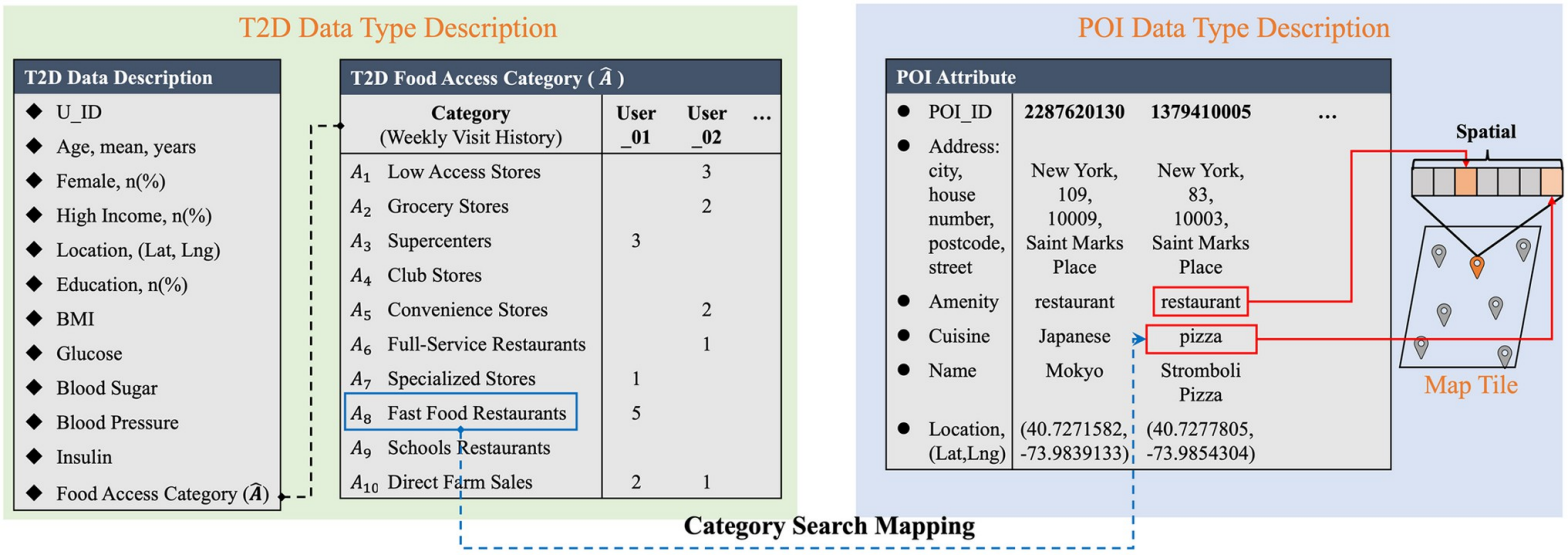
A schema database relationship. Which contains the description of T2D data types and POI data types.

Typically, the food access category corresponds to multiple POI attributes, while POI attributes are diverse and could be mapped by multiple categories. In Fig 2, we list the column name of the schema database, which contains the description of category and POI data types. We define the T2D food access category $\hat{A}=\left(A_{1},A_{2},A_{3},\ldots ,A_{10}\right)$ as an index into the table: T2D data description, where *A*_1_ to *A*_10_ correspond to the above 10 food access items, respectively. Food access data is the weekly number of Twitter user visits with categories, where the *user*_01_ has visited the fast food restaurant (*A*_8_) five times in a week. The visit category will be mapped to food access item-related POI attributes for training; thus, we list the utilized column name of the POI in data preprocessing. The POI attributes of food access items are labeled by category search mapping from the T2D case table to the POI table.

**Fig 3:**
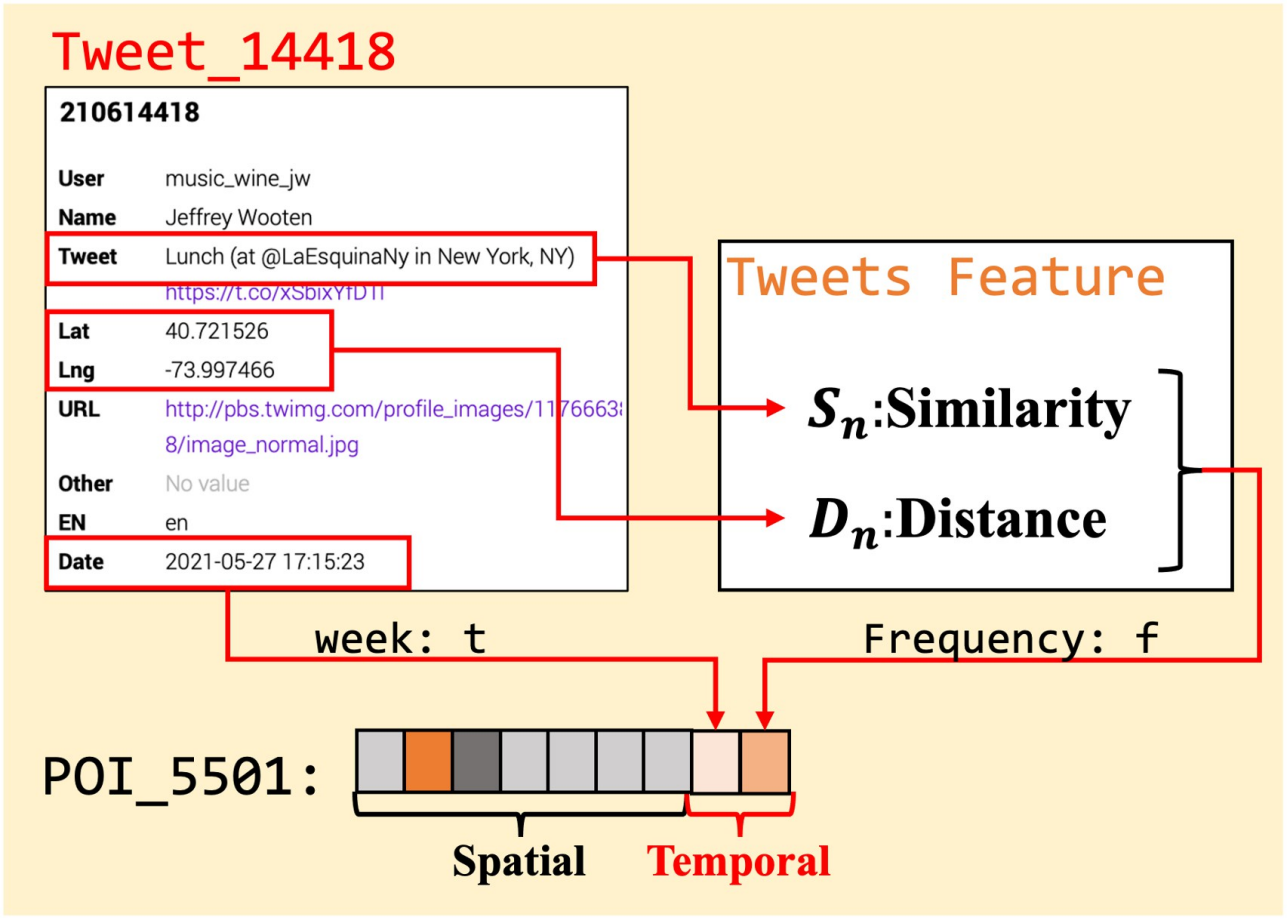
Example of temporal attributes and effective visit frequency for a given POI from a tweet record.

A set of geotagged Twitter records are utilized to build the dataset, and we complete the data preprocessing with the tweets examination. Each tweet is automatically generated using linked applications identified using regular expression matching and removed. These tweets generally contain the content posted by actual users, mostly showing messages such as “I’m at X restaurant” tweets, tweet coordinates match to the POI, and “a nice hamburger” food reviews. Combined with the coordinates and tweet check-in location in Fig 3, we map the “*Tweet*_14418_” to the “*POI*_5501_” as a piece of the user’s actual visit record. The Twitter user visit efficiency is denoted as the new attributes of relative POI, where *S*_*n*_ represents the place description similarity between the tweet *n* and POI attributes. *D*_*n*_ denotes the distance of the posted tweet position and the POI geolocation to remove POI relative tweets that cannot be counted in the same map tile. After preprocessing, POI and tweet data with a fixed format are used for the dataset.

## 5 Approach

In this section, we introduce the process of our proposed approach. Specifically, we first present the graph structure based on POI attributes on a set of map tiles that contain the related T2D case category. We discuss the training process of the spatial POI attributes and the mapping of the T2D food access category. Subsequently, we introduce the temporal POI attributes updating process by the dynamic tweet visit records and the LSTM module. Finally, we introduce the T2D risk prediction of the map tile regions by POI quantifying the social media visit data.

### 5.1 Feature splicing of tweets and POIs

**Fig 4:**
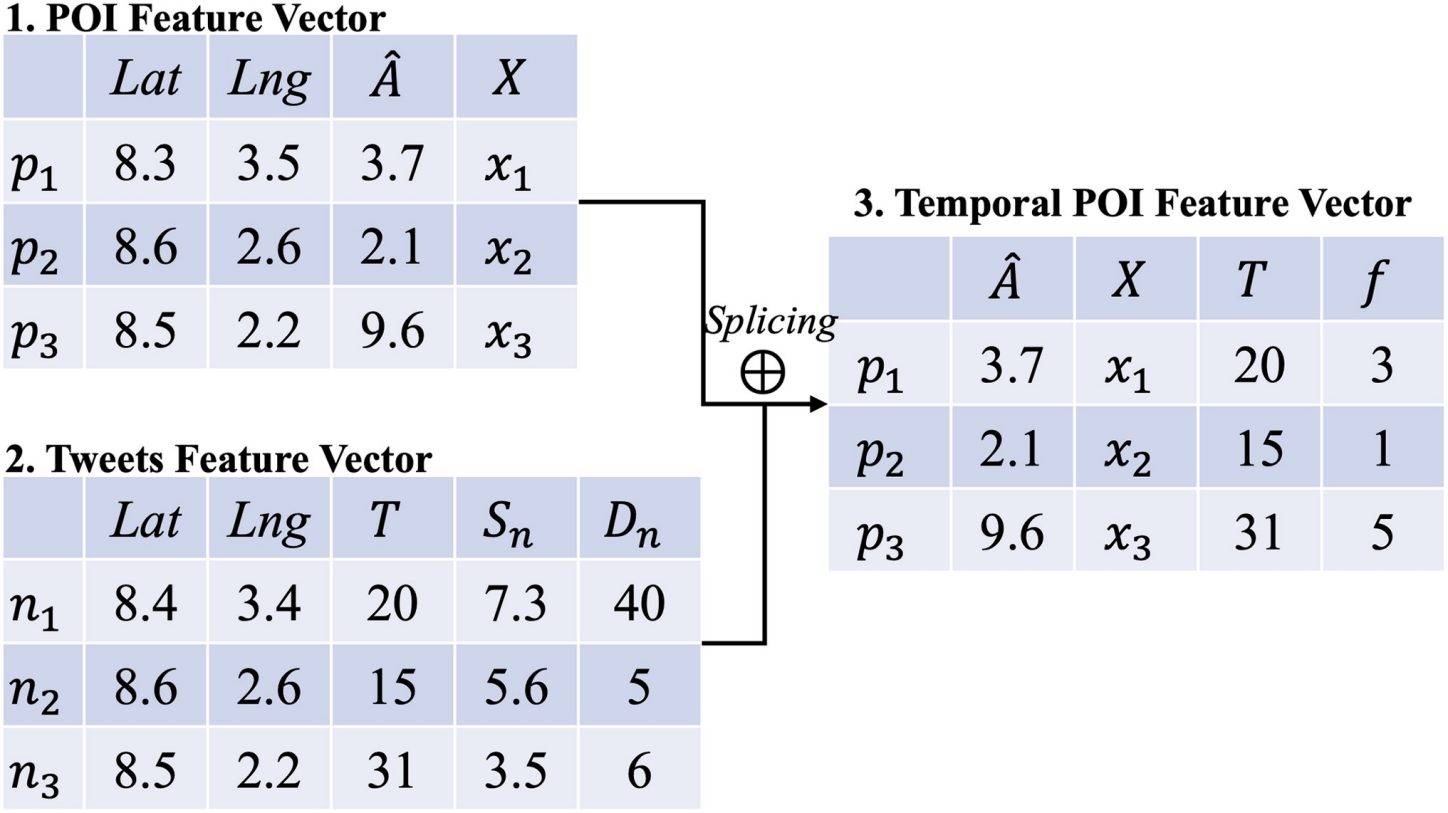
Feature splicing process of Tweets and POIs. *LatandLng*: coordinates, $\hat{A}$: category, *X*: node feature, *T*: time (T-th week), *S*_*n*_: similarity, *D*_*n*_: distance, *f*: visit frequency.

In Fig 1, we introduce the map tile graph construction and Twitter check-in data as the input before the process of the GLSTM approach block. Before the POI attribute extraction from T2D records with the GCN training model, the problem is that the POI location contains the spatial feature without the temporal feature of T2D food access. Therefore, we propose the feature splicing of POI attributes and Twitter check-in data, which confers the temporal attributes to the T2D food access visit features as the label ⊕ from Input Block to Tweets Extraction Block. In this subsection, we first introduce the feature splicing process from Twitter records input to the POI position attributes shown in Fig 4. The feature splicing process is the feature representation based on the POI location and Twitter user visit frequency, which contributes to the time series by the similarity and distance of POI attributes and tweet information.

In Fig 4, we list the POI feature vector that contains the coordinates (latitude and longitude), food access category $\hat{A}$, and graph structure information *X*. For the POI-related temporal attributes extraction process, we utilized coordinate search to map the tweets feature that has a similarity *S*_*n*_ with the POI description in the same map tile:

$$\begin{array}{c} S\left(n\right)=\frac{\sum_{i=1}^{n}p_{i}\times n_{i}}{\sqrt{\sum_{i=1}^{n}p_{i}^{2}}\times \sqrt{\sum_{i=1}^{n}n_{i}^{2}}}\times 100\%\times 0.1 \end{array}$$ (1)

where *p*_*i*_ denotes the description of POI and *n*_*i*_ is the tweet record. We standardize the feature vector labels, where the values of similarity *S*_*n*_, latitude, and longitude are the degrees between 0 and 10. A threshold is given for the similarity, in which only tweets of *S*_*n*_ > 3.5 are collected to count the user visit frequency.

In addition, we define the distance value *D*(*n*) between the tweet geolocation and the associate POI position as follows:

$$\begin{array}{c} D\left(n\right)=hav|d_{p_{i}}-d_{n_{i}}| \end{array}$$ (2)

Where $d_{p_{i}}$ and $d_{n_{i}}$ denote the position of the POI and the tweet, respectively; the calculation procedure *hav* refers to the haversine formula [27]. A threshold is provided for the distance, in which only tweet distances of *D*_*n*_ < 3(*km*) are collected to count the user visit frequency *f*:

$$\begin{array}{c} f{\left(P_{n}\right)}^{t}=\sum S\left(n\right)>3.5|\sum D\left(n\right)<3 \end{array}$$ (3)

After feature splicing processing, each POI contains the attributes denoted by T2D food access category $\hat{A}$, POI coordinates *lat*, *lng*, graph structure information *X*, the frequency *f* of Twitter user visits in week *T* from 2020 on wards is used to match the food access category of our data.

### 5.2 Map tile graph structure of GCN

**Fig 5:**
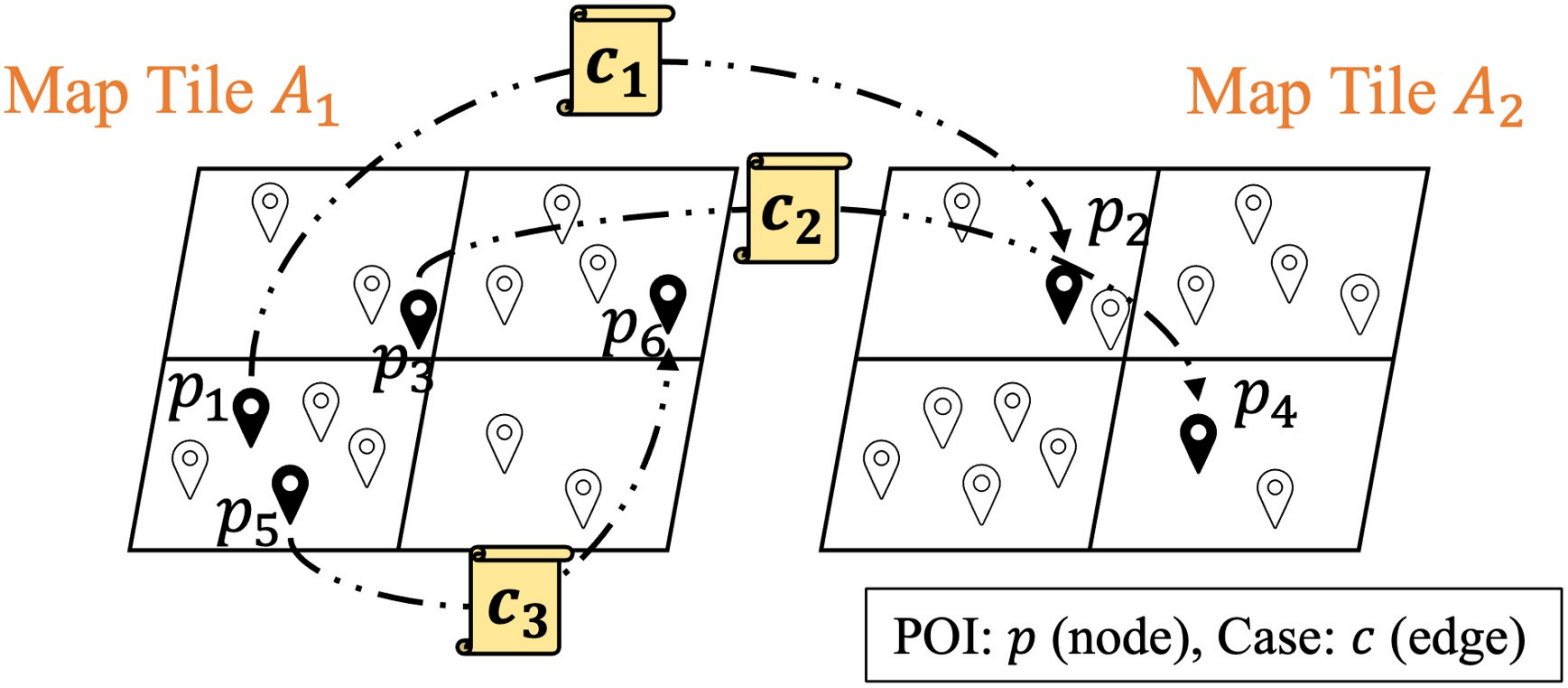
The graph structure on the map tile with different categories (*A*_1_ and *A*_2_). Where the POI denotes the node set and case categories are the edge information set of the GNN training.

In this subsection, we first discuss the processing of map tile map constructs, which is introduced in the Input Block of Fig 1. Considering the relationship between cases, POI attributes, and food access categories, we collect visit history from weekly food access of T2D patients via geolocation on OpenStreetMap tiles of Fig 5.

In Fig 5, we introduce the GNN graph construction on the map tile. A map tile *M* contains all food access categories $\hat{A}=\left(A_{1},A_{2},A_{3},\ldots ,A_{10}\right)$, the category relative POIs *p*_*i*_, and the category cases *c*_*n*_. The GNN graph construction of map tile $M_{\hat{A}}$ at each time point *t* ∈ [1, *T*] is $G_{M}^{T}=\left(p,c\right)$, where POI denotes the node set and the case contributes the edge information of the graph structure. We first introduce edge information processing to aggregate the POI attributes as the inputs of the GLSTM model. The POI node set *P*_*ij*_, contains all the POI subscript *p*_*i*_ to *p*_*j*_ on the map tile. In edge aggregation, we define the feature information of all nodes on the edge as aggregated for each edge information (T2D case category of this map tile). Regional information such as the T2D food access category relative features of the POI can be extracted from each map tile. For the aggregated POI *P*_*ij*_, we have the edge information of the category cases ${\vec{c}}_{n}$ [28]:

$$\begin{array}{c} {\vec{c}}_{n}=\frac{1}{K}\sum_{k=1}^{K}\sum_{N_{ij}}\alpha^{k}W^{c}{\vec{p}}_{ij} \end{array}$$ (4)

where *N*_*ij*_ is the set of category cases relative POI numbers, *W* is the trainable weight matrix, *K* is the number of heads attention, and *α*^*k*^ represents the attention coefficient calculated by the *k* − *th* attention mechanism. The attention coefficient *α* of the edge feature ${\vec{c}}_{n}$ and the corresponding aggregate POI feature $\vec{p}$ is:

$$\begin{array}{c} \alpha =\frac{exp(\sigma ({\vec{b}}^{T}[W^{c}{\vec{c}}_{n}\|W{\vec{p}}_{i}]))}{\sum_{N_{ij}}exp(\sigma ({\vec{b}}^{T}[W^{c}{\vec{c}}_{n}\|W{\vec{p}}_{ij}]))} \end{array}$$ (5)

where $\vec{b}$ is $W\vec{c_{n}}$, and *σ* is an activation function that adopts LeakyReLU. After each T2D case information of the edge is aggregated to the POI vectors, the aggregation process generates a new state to learn the T2D case visit category of $p_{A_{i}}$ to every POI node *p* of the GCN model. In Fig 5, we introduce the POIs of the T2D association categories *A*_1_ and *A*_2_ on two map tiles, respectively, where map tile *A*_1_ denotes the category *A*_1_ related POIs as black placemarks, and the category *A*_2_ related POIs with deep marks are represented in tile *A*_2_. We utilize this map tile visualization to determine the relationship of POIs in the same category and T2D case connection. We define each POI neighbor containing the aggregate information in proportion and representing the learnable attention coefficients as *N*_*p*_. For a single-layer GCN model, the new state *H* is convoluted as a signal matrix [29]; then, we have:

$$\begin{array}{c} H_{i}^{t}=\sigma (\sum_{p_{j}\in N(p_{i})}X_{p_{j}}W+b) \end{array}$$ (6)

where *W* is a parameter matrix for POI feature training, *b* is a learnable bias value, and *σ* is an activation function that adopts ReLU. *p*_*j*_ is the node set of node *p*_*i*_ neighbors. For the time point *t*, the POI with T2D case information convolutional process of the standard model is:

$$\begin{array}{c} H^{t}=\sigma ({\tilde{D}}^{-\frac{1}{2}}A{\tilde{D}}^{-\frac{1}{2}}X_{p}^{t}W) \end{array}$$ (7)

Given an adjacency matrix of POI attributes *A* and the feature matrix *X*, the GCN model constructs a filter in the Fourier domain as the first-order structural dependency relationships with a single layer. The filter acting on the POIs of a graph captures spatial features between the POI locations by its first-order neighborhood; then, the GCN model can be built by stacking multiple convolutional layers, which can be expressed as ([30]):

$$\begin{array}{c} H^{t(l+1)}=\sigma ({\tilde{D}}^{-\frac{1}{2}}A{\tilde{D}}^{-\frac{1}{2}}H^{t(l)}W^{\left(l\right)}) \end{array}$$ (8)

where *A* = *A* + *I*_*N*_ is the matrix with added self-connections, *I*_*N*_ is the identity matrix, $\tilde{D}$ is the degree matrix, $\tilde{D}=\sum_{j}A_{ij}$, *H*^*t*(*l*)^ is the output of the *l* layer at time *t*, and *W*^(*l*)^ contains the parameters of that layer.

In summary, we use the GCN model with the temporal parameters to learn spatial features from POI attributes with the T2D food access category data. As shown in Fig 5, such as the relation of *p*_3_ and *p*_4_ with *c*_2_, the GCN model can obtain the topological relationship of each POI combined with the food access category in the same map tile, encode the POI visit feature from T2D cases, and then obtain the spatial dependence.

### 5.3 Long short-term memory module

As Twitter users’ visits to and interactions with T2D food access category related POIs evolve over time, we believe that modeling the temporal dynamics is a key factor in accurately predicting early risk. After the POI feature is represented by the GCN model, we expect to utilize an important feature extraction module that can improve the performance of the T2D food access category risk prediction. Inspired by the success of related works for modeling sequential behavior data with recurrent neural networks (RNN), we utilize the LSTM module to capture the evolvement of dynamic T2D risk prediction based on Twitter user visit history.

**Fig 6:**
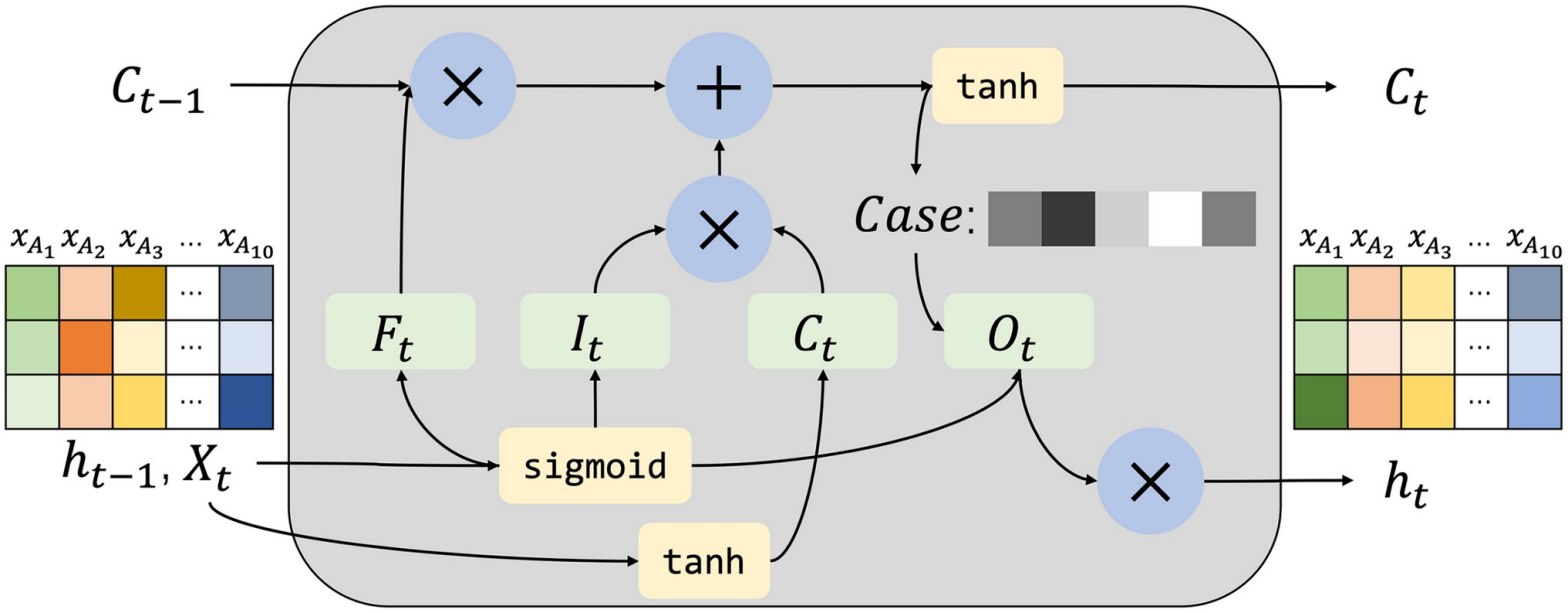
The structure of the LSTM module. Which contains the forget gate *F*_*t*_, input gate *I*_*t*_, cell sate *C*_*t*_, and output gate *O*_*t*_.

As shown in Fig 6, the input of the LSTM module contains the hidden state at time *t* − 1: *h*_*t*−1_, the spatial-temporal POI information at time *t*: *X*_*t*_, cell state at time *t* − 1: *C*_*t*−1_. The LSTM consists of 4 gates: forget gate *F*_*t*_ is to forget the information in the cell state selectively; input gate *I*_*t*_ determines what new information is stored in the cell state; output gate *O*_*t*_ determines what value we want to output; cell state *C*_*t*_ is the process of cell updating from the original *C*_*t*−1_ to the current *C*_*t*_. Finally, the LSTM module outputs the current hidden state *h*_*t*_, and cell state *C*_*t*_. The LSTM module obtains the POI features at time *t* by taking the hidden status at time *t* − 1 and the current POI features as inputs. While capturing the POI updating state at the current moment, the model still retains the changing trend of historical POI feature features from the food access attributes and has the ability to capture temporal dependence from Twitter user visit attributes.

The input for each time step of our proposed GLSTM framework is a combination of the original POI feature representations and the data representation output from the GCN network. We adopted a pairwise addition of these two data representations at each time point *t* ∈ [1, *T*]:

$$\begin{array}{c} {\text{X}}_{p}^{t}=\Delta {\text{X}}^{t}+\left(1-\alpha \right)H^{t(l+1)} \end{array}$$ (9)

where Δ is a hyperparameter used to balance the weight between the original T2D food access category related POI attributes representation $X_{p}^{t}$ and the spatial-temporal POI feature representation *H*^*t*(*l*+1)^ generated from the GCN. The transformation at each layer of the LSTM module is as follows:

$$\begin{array}{c} F_{t}=\sigma (W_{F}[{\text{X}}_{p}^{t},h_{t-1}]+b_{F}) \end{array}$$ (10)

$$\begin{array}{c} I_{t}=\sigma (W_{I}[{\text{X}}_{p}^{t},h_{t-1}]+b_{I}) \end{array}$$ (11)

$$\begin{array}{c} O_{t}=\sigma (W_{O}[{\text{X}}_{case}{\text{X}}_{p}^{t},h_{t-1}]+b_{O}) \end{array}$$ (12)

$$\begin{array}{c} C_{t}=F_{t}C_{t-1}+I_{t}[\text{tanh}(W_{g}[{\text{X}}_{p}^{t},h_{t-1}]+b_{C})] \end{array}$$ (13)

$$\begin{array}{c} h_{t}=O_{t}\;\text{tanh}\;C_{t} \end{array}$$ (14)

where *F*_*t*_, *I*_*t*_, and *O*_*t*_ are limited in the range from 0 to 1, which are used to filter the current information at a certain probability; *C*_*t*_ is the global cell state that enables the sharing of different cell outputs through the LSTM networks; *W*_*F*_, *W*_*I*_, *W*_*O*_, and *W*_*g*_ are trainable weight parameters for the LSTM module; *b*_*f*_, *b*_*I*_, *b*_*O*_, and *b*_*C*_ are denoted the trainable bias parameters. The transformation of *Sigmoid* and tanh are the activation functions, ⊗ denotes the vector transfer, and ⊕ denotes the vector concatenate.

In summary, the LSTM module in the proposed GLSTM framework completes the POI feature representation which can concentrate on the T2D case food access category of spatial dependence and Twitter user visit history-based temporal dynamics. On the one hand, the GCN model is used to compute the behavioral relationships between POI attributes of map tile-based geographic information and category cases to obtain spatial dependencies. On the other hand, the gated recurrent unit is used to capture dynamic changes in tweet visits frequency based on different times to obtain temporal dependence and to accomplish the T2D early risk prediction task.

### 5.4 T2D risk visualization

**Fig 7:**
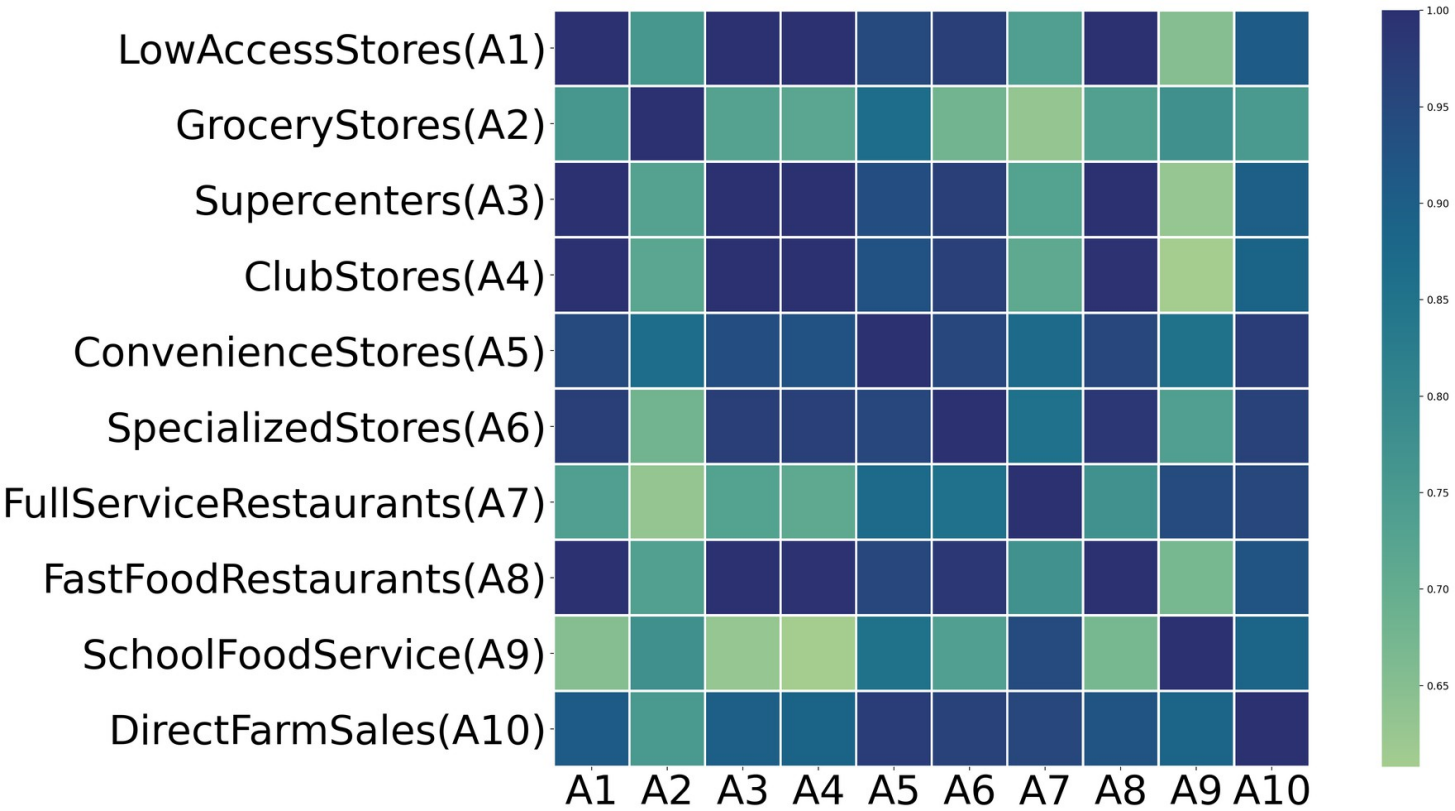
The T2D food access spatial weight matrix. Where the value of each grid is utilized to evaluate the matching degree of POI feature and category cases on the map tile.

In this subsection, we introduce the relationship between the output POI feature and categories with a spatial weight matrix shown in Fig 7, which is utilized to manage the early risk on the map.

The risk visualization process is designed to map the output POI features to areas highly similar to food access in category cases to illustrate the findings in relation to the disease. The matching degree between the Twitter user’s visit behavior of POI and the T2D case food access is quantified as a set of spatial vectors called correlation coefficients. Correlation coefficients can be computed by any correlation methodology that is suitable for the data structure, such as the Pearson method for linear correlations, the Spearman method for non-linear (rank) correlations, or polychoric/polyserial correlation methods for ordinal variables ([31]). Associated risk can be visualized as odds ratios (ORs) or beta coefficients from logistic or linear regressions in a forest plot. In this study, we adopt geographically weighted random forest (GW-RF) to discuss the spatial heterogeneity of T2D food access on the map tile [32]. The GW-RF model can improve predictive performance relative to a non-geographically weighted GNN model, which cannot resolve heterogeneous spatial processes.

The main idea of GW-RF is to calibrate locally rather than globally by integrating spatial weight matrix and random forest into a local regression analysis framework. For a map tile in our study, the spatial weight matrix is denoted as food access categories based on the POI feature with user temporal visited history. A spatial weight matrix: Fig 7 is composed of the T2D food access categories which are represented by the construction matching of POI attributes and category cases. The values of each grid in the category matrix are used to visualize the relationship between the frequency of user visits from Twitter check-in records and the food access category of T2D cases. Darker shades indicate that the user visit habits in the region to POIs are more consistent with the performance of T2D cases, which indicates a higher T2D early risk for susceptible people.

## 6 Evaluation

This section evaluates the performance of the proposed GLSTM training model using the social media dataset collected from Twitter in the US. We introduced the accuracy of the GLSTM prediction model on the Google base map and considered a case study of the New York State. The training process is as follows:

First, the POI displayed on 100 Google map tiles was used as the learnable benchmark. The relationship between the POI attributes, geolocation, and T2D case categories are shown in Fig 2. In addition, we set the weight of the food access category according to diabetes-related research [33].

Second, the POI attributes with the T2D case categories were trained by different models, including the variation algorithm of convolutional neural networks (CNN), GCN, and LSTM.

Third, the regional T2D risk visualization process was evaluated by the user visit data from Twitter records, as seen in Fig 1.

### 6.1 Dataset

We used the T2D cases and Twitter records with the geolocation on the map tiles, which included three domains: spatial locations, visit attributes, and temporal updates. The domains of the labeled POI include the following:

- **Spatial Location**: we collected 73,068 pieces of learnable POIs, which contain the coordinates and visit attributes on the open map: OpenStreetMap.
- **Visit Attributes**: we collected 198,377 tweets that match the POI food access visit attributes for training and evaluation.
- **Temporal Update**: contains the dynamic POI attributes update of Tweet access records as a time series.

The dataset was divided into training, validation, and test sets. We collected 2,031 cases from open resources in seven eastern US states. 136,472 tweet records and 58,375 POIs were utilized for a 70-tile training set; 61,905 tweets and 14,693 POIs were recorded in a 30-tile test set. A review set was used for validation by other 7,394 POIs.

### 6.2 Baselines

Our proposed method adopts the social media data to complete the POI feature splicing and utilizes a set of T2D food access cases to sample the POI attributes on spatial-temporal map tiles. We considered the geographical distribution of POIs on the map in the training groups and divided the dense POI core with roads as trainable map tiles. We compared our proposed GLSTM model with the baseline models including a convolutional neural network with LSTM (CNN-LSTM, 2018), convolutional and recurrent neural networks (CRNN, 2021), a temporal-spatial three-way recommendation model based on a recurrent neural network (RNN-TS3WR, 2022), and a graph neural network with bidirectional LSTM (GNN-BiLSTM, 2021).

- **CNN-LSTM** ([20]): A GCN variant model that improves on DSTG and considers the POI visit preference on the 3D maps. The semi-supervised node-partition strategy on a GCN provides cross-pixel correlations.
- **CRNN** ([34]): utilizes the convolutional and recurrent neural networks to develop a deep neural network architecture to predict diabetes progression and suggest disease management strategies.
- **RNN-TS3WR** ([35]): integrates the time factor with the space factor to realize the recommendation with lower decision costs. It adopts an RNN to extract the sequence of all previous records as inputs to predict the prescription efficacy when the current prescription is provided for each patient.
- **GNN-BiLSTM** ([36]): This focuses on the next POI recommendation task and the inclusion of user embeddings of global STP factors across all users to improve the recommendation task and user experience.

For a given test POI visit sample from Twitter users, if the food access category of POI obeys the T2D case behaviors of the Google Base Map set, then the accuracy has a positive value of 1; otherwise, it is 0. Further, we adopted the root mean squared error (RMSE) and the prediction recall based on Twitter data to evaluate the baseline models and GLSTM.

$$\begin{array}{c} RMSE=\sqrt{\frac{1}{n}\sum_{i=1}^{n}(a_{i}-f_{i}{)}^{2}} \end{array}$$ (15)

The RMSE value represents the predicted value and the sample standard deviation of the residual between the predicted values. RMSE describes the sample’s discrete results. In this study‘s nonlinear fitting training process, the smaller the value of the RMSE, the smaller the dispersion between the prediction results of the training model and the actual value, and the better the model performance.

The spatial-temporal regression performance score is the Twitter user visit recall of the training model’s POI attribute prediction as follows:

$$\begin{array}{c} Recall=\frac{TP_{tweets}}{TP_{tweets}+FN_{cases}}\times 100\% \end{array}$$ (16)

where *TP*_*tweets*_ is the count of positive tweet prediction based on the POI visit behaviors of T2D cases, and *FN*_*cases*_ is the negative POI prediction count of T2D food access category cases.

### 6.3 Results

We randomly selected 70 map tiles from the test set, where the tweet dataset is divided into test set A: 2020 and test set B: 2021 by 4 quarters or 13 weeks. We generated the Twitter user food access features of each POI using the GLSTM and baseline models.

Table 1 lists the performance of the training models. We compared the POI attribute processing ability of spatial-temporal regression and evaluated them using RMSE, prediction accuracy, and recall (tweet visits). The proposed method, GLSTM, outperformed the baseline models in temporal tweet-visit regression and spatial T2D risk prediction accuracy in two test sets.

**Table 1:** The spatial-temporal T2D food access category associated POI attributes prediction result with CNN-LSTM, CRNN, RNN-TS3WR, GNN-BiLSTM, and GLSTM training models.

Model	Metric	Set A, 2020 (Quarter/13weeks)					Set B, 2021 (Quarter/13weeks)				
		1st	2nd	3rd	4th	Ave	1st	2nd	3rd	4th	Ave
CNN-LSTM	RMSE	52.61	52.55	51.73	53.13	52.51	53.02	52.24	52.38	52.69	52.58
	Acc	71.08	68.49	69.36	68.44	69.34	69.92	69.19	68.02	68.27	68.85
	Recall	60.43	63.81	61.97	59.49	61.43	62.17	63.33	61.75	62.08	62.33
CRNN	RMSE	55.38	56.04	55.73	55.89	55.76	54.33	56.02	55.58	57.31	55.81
	Acc	73.01	71.36	70.28	72.66	71.83	71.18	72.05	70.77	72.02	71.51
	Recall	*	*	*	*	*	*	*	*	*	*
RNN-TS3WR	RMSE	53.26	55.84	56.71	54.38	55.05	54.88	53.05	55.89	56.18	55
	Acc	72.45	74.01	73.74	72.99	73.30	75.65	73.42	73.68	72.18	73.73
	Recall	63.75	64.08	63.37	63.18	63.60	62.95	62.74	61.13	63.33	62.54
GNN-BiLSTM	RMSE	64.75	62.16	62.58	61.93	62.86	63.27	62.75	63.11	62.58	62.93
	Acc	76.99	76.24	75.85	76.18	76.32	74.96	75.27	75.33	75.8	75.34
	Recall	65.08	65.73	64.88	65.17	65.22	64.26	65.29	64.96	65.81	65.08
GLSTM	RMSE	66.24	67.33	67.06	65.92	66.64	66.18	**68.24**	67.95	66.28	**67.16**
	Acc	**80.55**	79.38	79.09	80.36	**79.85**	78.16	79.51	80.03	79.94	79.41
	Recall	68.91	**69.52**	68.33	69.77	**69.13**	67.28	68.45	68.21	68.09	68.01

Specifically, the standard deviation of the residuals, RMSE, reflects the deviation of positive prediction results of high T2D risk POIs with the prediction errors based on social media visits. GLSTM achieved the best RMSE performance of 68.24% in set B and 67.33% in set A. The average RMSE performance in 2020 of set A, GLSTM model has a highly improvement from 52.51% of CNN-LSTM, to 66.64%, and in 2021 of set B, GLSTM improved from 52.58% of CNN-LSTM, to 67.16%.

The values of prediction accuracy (“Acc”) reflect the positive prediction results of T2D food access categories related to POIs by users’ social media visits. The best accuracy prediction performance is 80.55% for GLSTM in set A, which is improved from 71.08% of the CNN-LSTM model. In set B, GLSTM also achieved the best prediction performance of 80.03% in the third quarter of 2021. When combining the average prediction accuracy results, GLSTM has the best performance 79.85% in set A and 79.41% in set B.

The values of “Recall” denote the spatial-temporal POI attribute’s regression performance based on the Twitter user visit history of the T2D category predictive results. Here, the proposed GLSTM model obtained the best results of 69.52% in the second quarter, 2020 in set A and 69.13% in average results, which is better than 61.43% of the CNN-LSTM model. In set B, GLSTM achieved the best recall performance of 68.45% on the second quarter of 2021 and 68.01% in average results, indicating that it can adequately capture temporal dependence.

In addition, as a mature spatial-temporal regression method, the prediction results of the CRNN model are relatively lower than those of the RNN-TS3WR and GNN-BiLSTM models. The CRNN model has a set of non-stationary values, and the values are small enough to be negligible. Therefore, we use the label “*” to indicate that the model’s prediction effect is poor. We can see that the GLSTM model obtains the best prediction performance under most evaluation metrics for all prediction horizons, proving the effectiveness for spatiotemporal T2D food access-related POI attributes regression tasks.

The collected T2D cases with the food access category are utilized to address early T2D health management with timely monitoring of the susceptible population’s diet structure. Our proposed method is based on web geographic data mining and spatial information statistics, along with the discrete weekly temporal data distribution features composed of tweets. In Table 2, we list the differences in T2D food access category training performance in 7 US states, and the T2D food access risk prediction results denote differences in prediction accuracy from different states.

**Table 2:** The prediction results of our proposed GLSTM training model with the T2D food access categories in 7 States.

Code State	FL Florida	GA Georgia	VA Virginia	PA Pennsylvania	NJ New Jersey	NY New York	MA Massachusetts
Our T2D Cases	304	255	316	251	270	353	282
Our Collected POIs, n*1000	11.12	9.65	12.84	8.97	8.06	15.65	6.71
Our Collected Tweets, n*1000	27.58	26.49	32.09	26.35	25.61	35.73	24.55
Age,mean,years	43.07	45.62	44.25	42.96	43.33	45.17	43.72
HighIncome, Incomplete, n(%)	54.33	55.92	55.08	53.75	54.66	61.25	59.28
Food Access, week,(* times)							
A1 LowAccessStores	288	341	309	297	330	384	318
A2 GroceryStores	254	230	208	271	284	337	267
A3 Supercenters	455	372	399	383	408	427	380
A4 ClubStores	138	143	125	153	144	198	127
A5 ConvenienceStores	472	431	428	395	419	496	408
A6 SpecializedFoodStores	203	195	155	167	138	237	169
A7 Full-ServiceRestaurants	152	128	133	115	136	203	140
A8 Fast-FoodRestaurants	427	455	430	395	406	472	436
A9 SchoolFoodService	58	74	36	65	75	85	74
A10 DirectFarmSales	173	142	137	159	202	253	206
RMSE(GLSTM)	67.38%	66.25%	**68.08%**	67.05%	66.08%	68.06%	66.33%
Prediction Accuracy (GLSTM)	78.37%	79.28%	81.25%	77.33%	75.29%	**83.26%**	73.26%
Recall (Twitter)	69.62%	67.55%	68.77%	68.31%	67.94%	**70.32%**	68.27%

Compared with the average RMSE value of 66.90% from the GLSTM model, the RMSE values of the states of New York, Virginia, Florida, and Pennsylvania are 68.06%, 68.08%, 67.38%, and 67.05%, respectively; all these values are greater than the average value. Therefore, we focus on the results of the GLSTM model in these four states. Specifically, the results of New York had the best prediction accuracy of 83.26% and recall value of 70.32%. Virginia shows the best RMES performance of 68.08%, but the accuracy of 81.25% and recall of 68.77% is lower than that of New York. The accuracy results of Florida and Pennsylvania are 78.37% and 77.33%, respectively, better than New Jersey and Massachusetts; however, they are still lower Virginia and New York. As for the GLSTM model’s recall value from Twitter data testing, Florida’s performance of 69.62% is better than 68.31% of Pennsylvania and 68.77% of Virginia, but lower than 70.32% of New York.

Considering the average difference of these four states’ results, we investigated the data that was collected within 7 states, where New York, Virginia, and Florida have the highest number of POIs, 15.65, 12.84, and 11.06 thousand, respectively; These states also had the highest number of tweets, 35.73, 32.09, and 27.58 thousand, respectively. For the collected T2D cases, New York and Georgia have higher mean ages, and New York and Massachusetts have higher incomes. Overall, the differences in the sample sizes of POI and tweet data and the sample of the T2D case income information are responsible for the differences in the evaluation results of the GLSTM model across the states. The collected T2D cases with food access categories do not have enough geographic information and spatial features to allow the gradient of our training model to reach the threshold, resulting in unsatisfactory prediction accuracy and RMSE.

## 7 Case study

**Fig 8:**
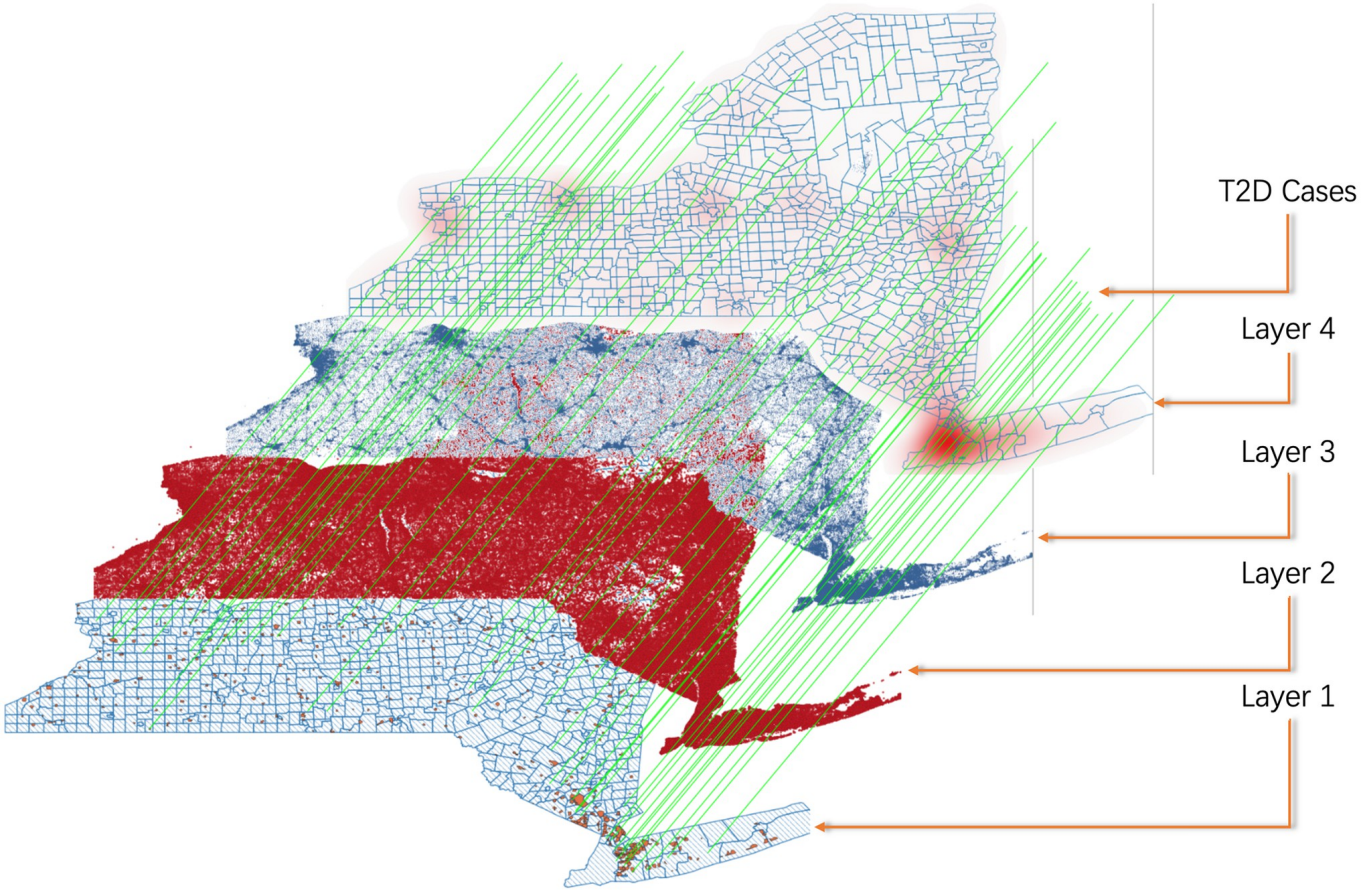
A T2D risk prediction based on a case study of the New York State map visualization.

To clearly discuss the process of food access category related risk visualization on the map combined with the POI attributes in the T2D case, we selected New York State in the results as a case study. For the discrete spatial data in each map tile, the prediction is given by the approximate predictive density method for parameter points combined with the T2D attributes and population food access features. In this section, we introduce the discrete T2D risk distribution of diabetes attributes in the food access feature, combined with a set of T2D cases and food access category related POI attributes, we utilize the heat map to visualize the early T2D risk prediction results by our proposed GLSTM model in Fig 8. The prediction results are visualized in four map layers, which contain the boundary map, tweet location, POIs with food access category, and heat map with T2D risks from bottom to top; T2D cases are mapped as green threads on the maps.

The first layer of the map visualizes the boundaries that contain states, counties, cities, towns, and villages of New York. This layer helps us in classifying T2D cases with the city population and base map tile segmentation. The boundary information in this layer is also utilized in the final visualization of the prediction results.

The second layer with red tweet places reflects the Twitter user gathering geolocations, which are utilized to visualize the key population distribution of each map tile and support us in classifying the temporal POI information with different food access categories. Each tweet record contains the coordinates, POI similarity, distance, and temporal information. In addition, each tweet feature denotes the user’s visited frequency of the related POI features, which are imported into the LSTM module of the proposed GLSTM model.

The third layer with blue POI marks is collected from OpenStreetMap, which contains the id, latitude, longitude, map tile number, address, amenity, cuisine, and store name. In this layer, we utilized the proposed GLSTM model to train the POI spatial feature with the T2D case food access category and update each POI temporal feature combined with the frequency of weekly tweet visit information from Layer 2.

The fourth layer is the heat map visualization of the POI early risk prediction results by the T2D case of food access categories. Here, the red regions represent a higher T2D susceptible incidence risk trained by the GLSTM model with T2D cases mapping. The map tile sets with the convolution kernel in the GLSTM training model identified and classified the T2D food access-related POI placemarks. Combining the Twitter data, we utilized the LSTM module to visualize the dynamic T2D food access relation of the POIs on the map. The weight distribution from the spatial-temporal POI features with T2D food access categories are utilized in the original features discussion, changing the original feature distribution and enhancing the effective features to suppress ineffective features or noise.

## 8 Conclusion

In this study, we collected 73,068 POIs on 100 map tiles, which were utilized to predict the early T2D risk with food access category and geographic features by the proposed spatial deep learning model. In addition, we collected 198,377 geotagged tweets in 2020 and 2022 to enhance the temporal feature of the LSTM module in the GLSTM framework. We added the POI geographical attributes associated with diabetes into a bag-of-words model and mapped them to the convolutional block attention module to predict the food access structured features.

According to the analysis of the data set, we summarized the POI attributes of T2D categories. The age of T2D patients is unevenly distributed from 16 to 76, which is the reason we considered the category of school food service. However, when combined with the data in Table 2, school food service has only a small proportion in our data, and we will continue to investigate the student case of T2D performance in the future. In contrast, the category of fast food, supercenters, and convenience stores have the highest percentages of food acquisition in each state, and access to high-calorie foods is also one of the major factors that directly increase the BMI index and affect the early risk of T2D.

In this study, we compared the results of the GLSTM model with other baselines; the proposed GLSTM model improved the prediction accuracy of 69.34% to 79.85%, indicating the effectiveness of spatial-temporal POI feature prediction. Furthermore, the GLSTM model achieved the best risk prediction results in New York. The visualization of the T2D risk category distribution can be used to provide more spatially scalable risk predictions to observe the distribution of patients and susceptible populations in different regions. Our model provides a weekly, near real-time record analysis to promote model learning efficiency and rationalize diet structure management. In the future, we will focus on the application of cross-disease attribute analysis using geographic information.
